# Gaze data of 4243 participants shows link between leftward and superior attention biases and age

**DOI:** 10.1007/s00221-024-06823-w

**Published:** 2024-03-31

**Authors:** Christoph Strauch, Alex J. Hoogerbrugge, Antonia F. Ten Brink

**Affiliations:** Experimental Psychology, Utrecht University, Helmholtz Institute, Heidelberglaan 1, Utrecht, 3584CS The Netherlands

**Keywords:** Spatial attention, Pseudoneglect, Aging and attention, Asymmetry

## Abstract

Healthy individuals typically show more attention to the left than to the right (known as pseudoneglect), and to the upper than to the lower visual field (known as altitudinal pseudoneglect). These biases are thought to reflect asymmetries in neural processes. Attention biases have been used to investigate how these neural asymmetries change with age. However, inconsistent results have been reported regarding the presence and direction of age-related effects on horizontal and vertical attention biases. The observed inconsistencies may be due to insensitive measures and small sample sizes, that usually only feature extreme age groups. We investigated whether spatial attention biases, as indexed by gaze position during free viewing of a single image, are influenced by age. We analysed free-viewing data from 4,243 participants aged 5–65 years and found that attention biases shifted to the right and superior directions with increasing age. These findings are consistent with the idea of developing cerebral asymmetries with age and support the hypothesis of the origin of the leftward bias. Age modulations were found only for the first seven fixations, corresponding to the time window in which an absolute leftward bias in free viewing was previously observed. We interpret this as evidence that the horizontal and vertical attention biases are primarily present when orienting attention to a novel stimulus – and that age modulations of attention orienting are not global modulations of spatial attention. Taken together, our results suggest that attention orienting may be modulated by age and that cortical asymmetries may change with age.

**Supplementary Information:**

The online version contains supplementary material available at 10.1007/s00221-024-06823-w.

## Introduction

Healthy people typically show more attention to the left than to the right, and superior to inferior. These attention biases are thought to reflect horizontal and vertical asymmetries in neural processes. Changes in spatial bias with age are therefore likely to reflect age-related reorganisation of brain regions involved in spatial processing. These spatial attention biases are not only of fundamental interest, but are also important as a diagnostic feature of neurological conditions such as visuospatial neglect after stroke. It is therefore important to understand the effects of healthy aging on attention biases. While there is general agreement on the importance of this question (Friedrich et al. 2018; Jewell and McCourt 2000; Learmonth and Papadatou-Pastou 2021), the results obtained to date are highly inconsistent. This is likely due to task insensitivity and biases introduced by explicit overt responses, as well as small sample sizes or samples that include only very young or old age groups. In the current study, we addressed these shortcomings by analysing gaze data during free viewing of a single image by 4,243 individuals. This approach allowed us to study age effects on attention biases in a quasi-continuous manner.

Neurologically healthy controls show a leftward attention bias, as evidenced, for example, by deviated line bisection (Higier 1892; Jewell and McCourt 2000). This has been termed ‘pseudoneglect’ because of its similarity to the rightward bisection bias shown by people with visuospatial neglect following brain lesions (Bowers and Heilman 1980). A similar leftward bias in neurologically healthy controls is found in a number of other visuospatial tasks, such as greyscales (Nicholls et al. 1999), cued target detection (Heilman and Van Den Abell 1979), temporal order judgements (Pérez et al. 2008), and pupil responses (Strauch et al. 2022). Different tasks tap into different mechanisms of pseudoneglect, and a dissociation can be made between perceptual judgements and visual exploration (Chen et al. 2019). The leftward attention bias is most commonly attributed to the dominant role of the right hemisphere in visuospatial processing, known as the “activation-orientation hypothesis” (Benwell et al. 2014; Bultitude and Aimola Davies 2006; de Schotten 2005; Reuter-Lorenz et al. 1990). Other factors that may modulate, but do not fully explain, the leftward bias include habitual reading direction, handedness, and which hand is used for a given task (Friedrich and Elias 2014; Jewell and McCourt 2000).

Because a leftward attention bias is thought to reflect lateralization and hemispheric imbalances, it can be used to index these phenomena. For example, the development of attention bias with age has been used to investigate how hemispheric imbalance develops across the lifespan. A change in horizontal attention bias with age is to be expected, as there is evidence for a reduction in the asymmetry of hemispheric activity with age (Cabeza 2002; Cabeza et al. 1997). Furthermore, the right hemisphere is thought to age more rapidly than the left hemisphere, as suggested by the right hemi-aging model (Goldstein and Shelly 1981). The effect of age on horizontal bias has been studied extensively, but with inconclusive results thus far. Jewell and McCourt (2000) and Learmonth and Papadatou-Pastou (2021) reported that biases tend to shift to the right with age, while a systematic review by Friedrich et al. (2018) found that biases became either more leftward, neutral, or rightward, depending on the task used. This suggests that factors beyond purely visuospatial biases may be involved in the reported age-related biases. Visuomotor biases, response biases, or any other effects of specific task demands should be excluded from evaluations unless clearly marked as such. Therefore, measures that do not require overt responses such as pressing a button are likely to be superior. One promising measure is gaze position. Initial evidence from Chiffi et al. (2021) suggests a decrease in leftward gaze bias in free viewing with age in a sample of 60 participants.

In addition to the leftward bias, healthy controls show a *superior* bias on vertical versions of line bisection (Drain and Reuter-Lorenz 1996; Post et al. 2006; Shelton et al. 1990; van Vugt et al. 2000; Wolfe 1923) and greyscales tasks (Heber et al. 2010; Nicholls et al. 2006; Yamashita 2023), which is sometimes referred to as ‘altitudinal pseudoneglect’. This bias has been less studied and reported on than the horizontal bias and is arguably less understood. One hypothesis interprets the superior bias as another consequence of right hemispheric dominance in visuospatial tasks. This is based on observations of right posterior parietal lobe activation during both horizontal and vertical bisection tasks (Fink et al. 2001). Similar enhancements of leftward and superior bias with increasing cognitive load (Ciricugno et al. 2021) and presentation of the lines in the left hemispace (Suavansri et al. 2012) provide evidence for the idea of overlapping mechanisms. In contrast, horizontal and vertical bisection errors appear to be uncorrelated (Churches et al. 2017; Nicholls et al. 2004; van Vugt et al. 2000; but see Chieffi et al. 2019). The mechanisms underlying the reported horizontal and vertical asymmetries in attention may be idiosyncratic. This is because natural scenes in the world typically exhibit systematic asymmetry along the vertical, but not the horizontal, plane with respect to the most informative aspects of visual information. These known regularities about the world are likely to influence attention (Langley and McBeath 2023). In summary, while the leftward and superior biases may share some underlying mechanisms, different mechanisms may contribute equally (see also Drain and Reuter-Lorenz 1996; Silson et al. 2018).

The relationship between the superior bias and age has been less studied, and the reported effects of age on superior bias are similarly inconsistent. While superior biases in line bisection have been observed in both younger children (van Vugt et al. 2000) and older adults, with a stronger superior bias with increasing age (Mańkowska et al. 2018), no differences have been found between younger and older adults in the greyscales task, although this may be due to limited sensitivity (Yamashita 2023). In summary, it is unclear whether superior bias changes with age, due to task inconsistency and small sample sizes, which often include extreme rather than continuous age groups.

Gaze data can provide a sufficiently sensitive method for investigating attention biases: Leftward and superior biases are consistently reflected in gaze patterns and cannot be the result of explicit overt responses. Healthy young adults show a leftward gaze bias when freely viewing natural scenes. Perhaps due to a strong role of attentional orienting in pseudoneglect, the first saccade is more often leftward (Dickinson and Intraub 2009; Foulsham et al. 2013, 2018). This leftward bias peaks around the second to third fixation and persists for up to 1.5 s of a trial (Chiffi et al. 2021; Foulsham et al. 2018; Hartmann et al. 2019; Ossandon et al. 2014). This leftward gaze bias is robust to stimulus content (Ossandon et al. 2014), viewing distance (Hartmann et al. 2019), and even task goals such as visual search or memorization (Nuthmann and Clark 2023; Nuthmann and Matthias 2014; Zelinksy 1996). Furthermore, the bias remains present when there is no need to maintain central fixation before image onset, ruling out explanations related to asymmetries in fixation control (Ossandon et al. 2014). A *superior* gaze bias has been reported in visual search with only four observers (Zelinksy 1996), and a higher probability of upward than downward saccades has been described by Greene et al. (2014).

**Fig. 1:**
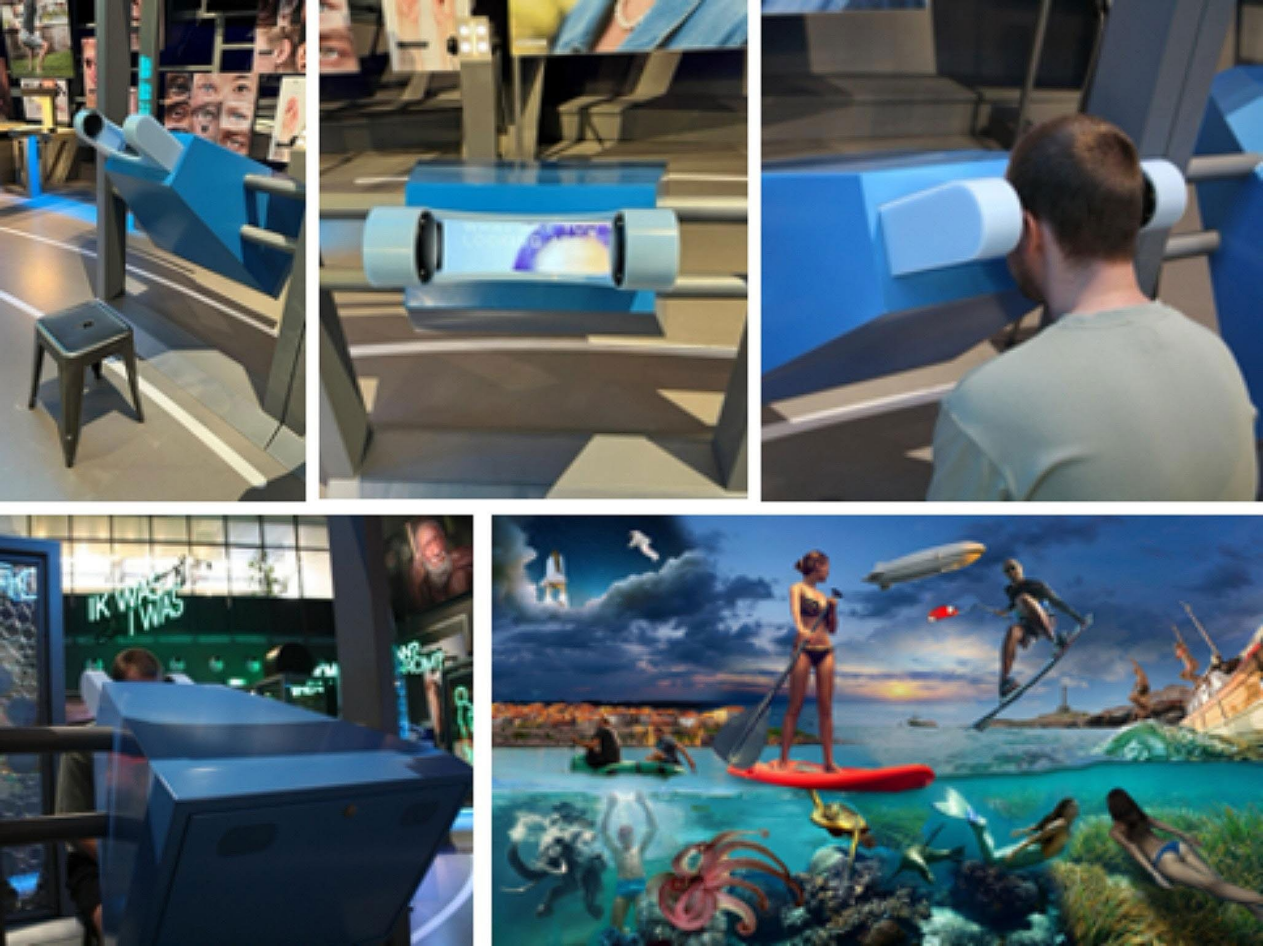
Setup (top row, bottom left) and stimulus (bottom right). The figures are reproduced/rearranged from Strauch et al. (2023). The stimulus was composed of licensed stock images from Shutterstock

In summary, gaze data may provide the optimal measure to study whether and how strongly age affects horizontal and vertical attention biases (Chiffi et al. 2021). However, such studies would require at least hundreds of individuals to provide robust and quasi-continuous estimates of how age modulates attention biases. As eye-tracking is generally expensive, such datasets are scarce. Here, we used data from a unique sample of 4,243 individuals aged 5 to 65, all of whom viewed a single image for 10s (setup and stimulus described in Strauch et al. 2023; Fig. 1). This allowed, for the first time, quasi-continuous estimates of horizontal and vertical biases across much of the lifespan in a large sample. We hypothesized that the leftward and superior biases in healthy participants, as reflected in gaze position during free viewing, would change with age.

## Methods

### Dataset

Gaze data were re-analyzed using the dataset described in Strauch et al. (2023). In short, visitors to the NEMO museum in Amsterdam viewed a single image for 10s before being given feedback on their eye movements, asked to donate their data and, if they agreed, to provide their age and gender. The image was presented on a 27”, 1920 × 1080 px monitor (50 × 24 degrees of visual angle) with a maximum luminance of 300 cd/m2, at 80 cm from the eyes to the screen. A Tobii 4 C eye tracker (a low-cost commercial tracker with a research license) was installed under the monitor to track the participants’ gaze at 60 Hz. A metal box around the monitor and eye tracker shielded the view to the sides and the relatively small opening ensured that the horizontal and vertical head position was central relative to the stimulus. To look into the metal box and participate, visitors could either stand, sit on a stool positioned next to the installation, or stand on the stool (see Fig. 1 for setup and stimulus). Gaze position was calibrated with a custom five-point calibration before stimulus onset. No instruction was given to participants, but participants were aware that their eyes would be tracked. Precision was calculated as in Hooge et al. (2018) and is given together with data loss in Supplementary Fig. 1. For further details, see Strauch et al. (2023).

**Fig. 2:**
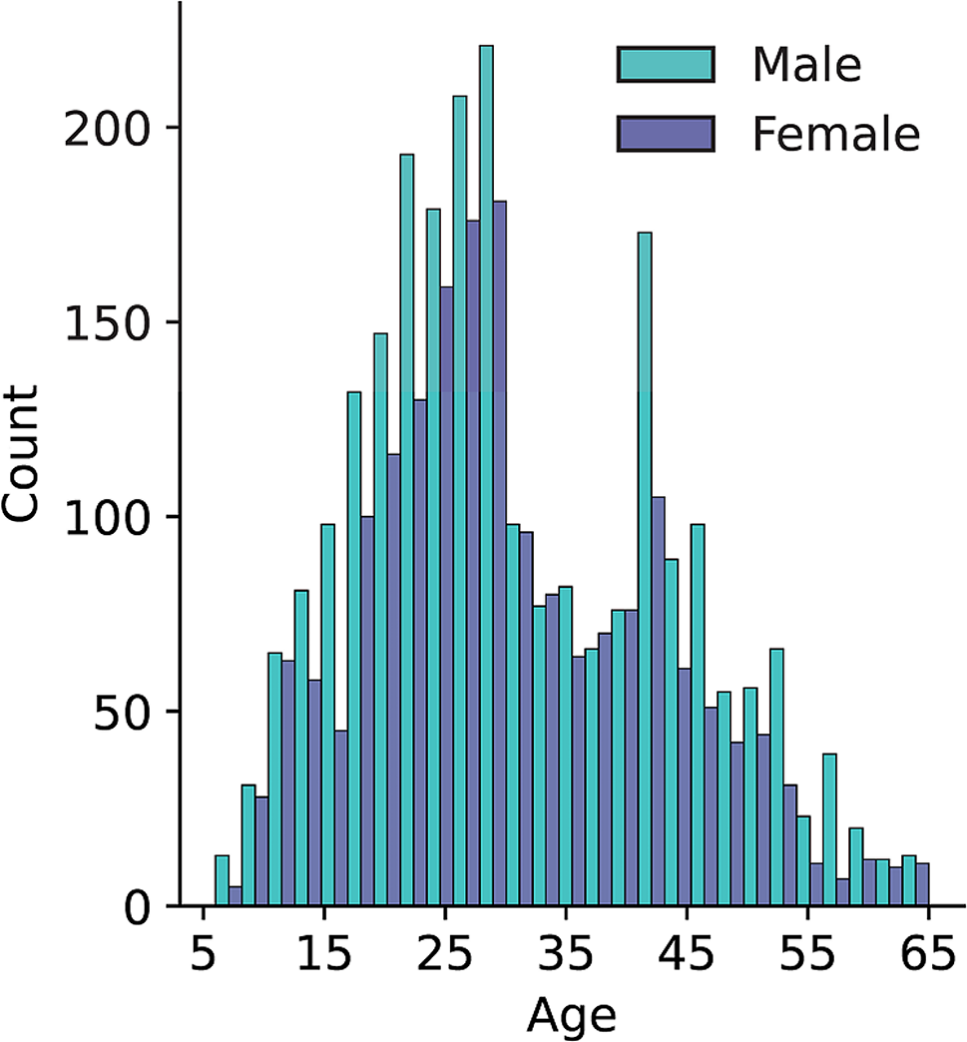
Number of participants across age for male (*n* = 2,411) and female (*n* = 1,832) participants. *Note* that data of participants with indicated year of birth as 2000 or non-binary gender could not be reliably analyzed and were therefore excluded, see Strauch et al. (2023)

Here, we considered data from all participants aged between 5 and 65 years and with at least 9 fixations (ca. 4 s of free viewing), resulting in data from 4,243 participants (*M* = 30.73 years, *SD* = 12.22 years; male: *n* = 2,411, female *n* = 1,832; note that data marked as non-binary have to be ignored due to a problem with the setup, see Strauch et al. 2023). Using only the first 9 fixations allowed the maximum number of participants to be included in the analyses to maximize statistical power. Figure 2 shows the number of participants per year of age, separately for reported gender.

Compiled data and analysis scripts are available via the Open Science Framework: https://osf.io/3dsr5/. Original data and preprocessing scripts are available via https://osf.io/sk4fr/ (Strauch et al. 2023).

### Data processing and analyses

We used the compiled fixation data from the original paper, removing all initial fixations that started before image onset. Note that participants did not always start in the center of the screen, so the data should not be interpreted regarding absolute gaze bias, but only its modulation with age. Next, we calculated gaze biases from the center per participant per fixation in both vertical and horizontal directions. We then averaged these biases across participants per year of age and per fixation. Spearman correlations were calculated to test for associations between gaze biases and age. Importantly, due to the non-symmetric nature of the image and the aforementioned differences in starting points, we did not test for the presence of *absolute* horizontal or vertical gaze biases but focused on the relative difference between people of different ages.

**Fig. 3:**
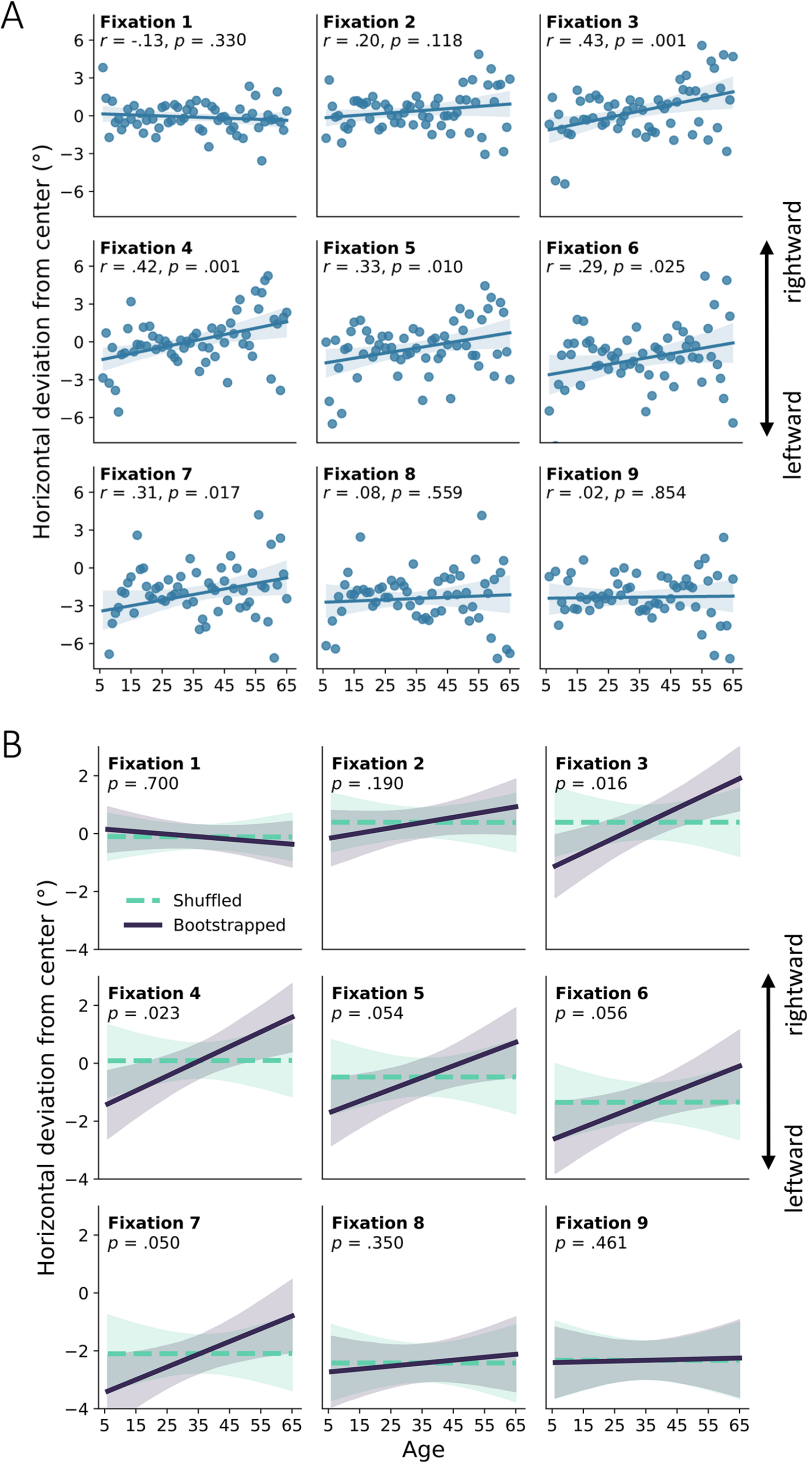
**A** Mean horizontal deviation of gaze position (left-right relative to the screen center) in degrees of visual angle across age, depicted for each of the first nine fixations. For illustration, straight lines indicate fitted linear regressions, shaded areas indicate 95% confidence intervals of these regressions; dots represent average positions per year of age. Gaze position was significantly modulated by age for fixations 3 to 7, with higher age associated with more rightward gaze position. **B** Bootstrapped regressions with 20 participants per age group (drawn with replacement) over 10,000 folds (solid purple line) against regressions with randomly shuffled age labels (dashed turquoise line). Shaded areas indicate the 95% range of bootstrapped values. Gaze position was significantly modulated by age for fixations 3, 4, and 7, with higher age associated with more rightward gaze position

**Fig. 4:**
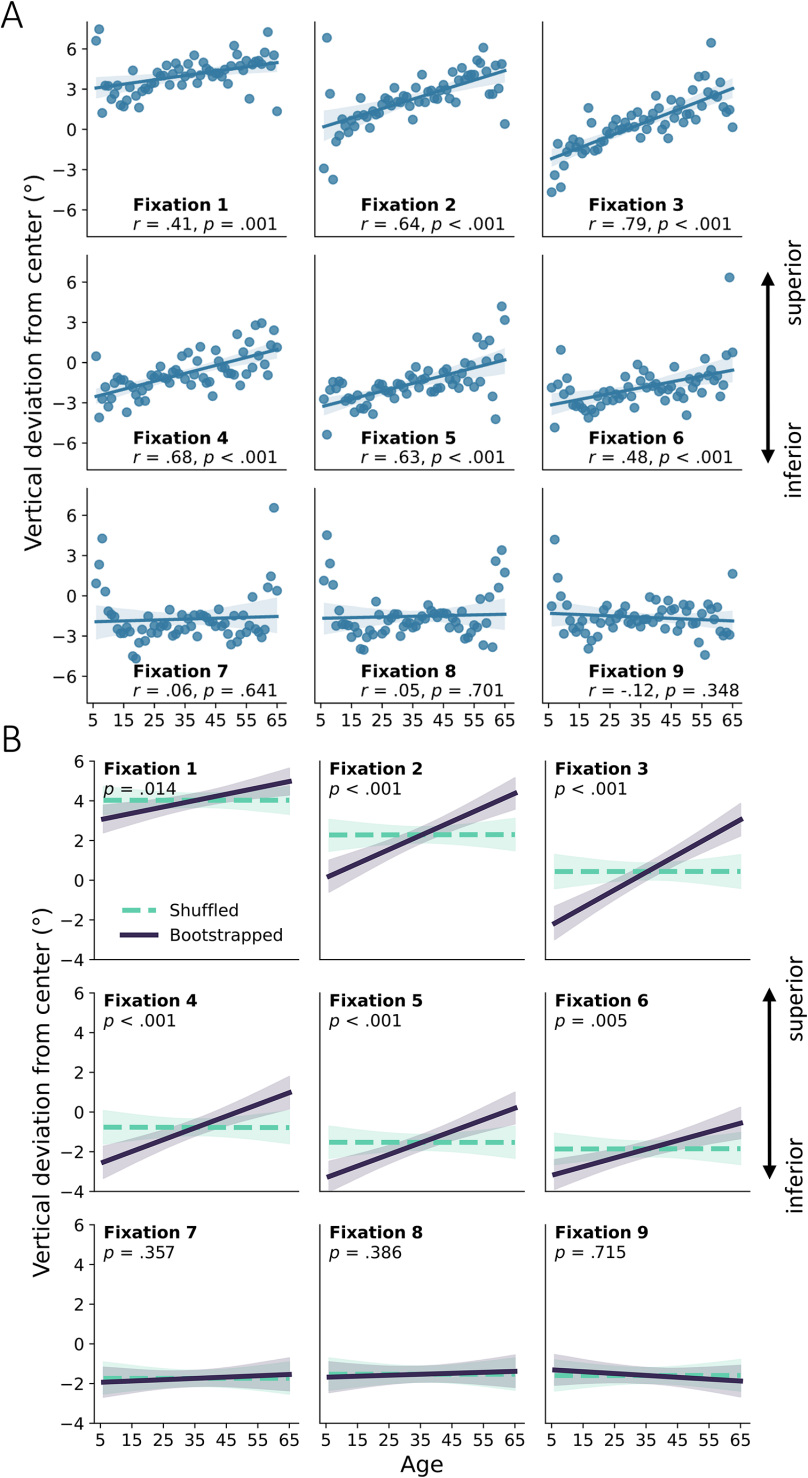
**A** Mean vertical deviation of gaze position (bottom-top relative to the screen center) in degrees of visual angle across age. For illustration, straight lines indicate fitted linear regressions, shaded areas indicate 95% confidence intervals of these regressions; dots represent average positions per year of age. Gaze position was significantly modulated by age for fixations 1 to 6, with higher age associated with more superior gaze position. **B** Bootstrapped regressions with 20 participants per age group (drawn with replacement) over 10,000 folds (solid purple line) against regressions with randomly shuffled age labels (dashed turquoise line). Shaded areas indicate the 95% range of bootstrapped values. Gaze position was significantly modulated by age for fixations 1 to 6, with higher age associated with more superior gaze position

Age groups were differently large (see Fig. 2), and as such, participants in age groups with fewer participants gave more weight to the analysis compared to participants in groups with more participants. To compensate for these possible biases, we computed regressions from bootstrapped data as a control analysis. For each age group, 20 participants were sampled with replacement, and a regression was computed for that subsample. This procedure was iterated 10,000 times, and we reported the average regression slopes and 95% ranges of obtained values in Figs. 3B and 4B (the distributions of correlation coefficients are reported in Supplementary Fig. 2). To test whether the obtained regression slopes were significantly different from chance, the age labels were shuffled in each iteration of the bootstrap procedure, and a regression was computed on this shuffled subsample of data. We then performed pairwise comparisons between the bootstrapped regressions and the shuffled regressions and report the proportion of iterations in which the bootstrapped regression slopes were smaller than or equal to the slopes of the shuffled data. This value provides an estimate of the chance that the obtained regression slopes are coincidentally greater than slopes obtained from randomized data, and is thus reported as a *p*-value.

## Results

To investigate a possible modulation of gaze bias with age, we correlated mean gaze biases in degrees of visual angle from the center in both horizontal and vertical dimensions for the first nine fixations with age (see Table 1 for the full statistics). Age was consistently associated with a more rightward gaze position for fixations three to seven (see Fig. 3A for scatterplots per fixation), with a maximum correlation at moderate effect size for the third fixation (*r* = 0.43, *p* = 0.001). Age and horizontal gaze position did not correlate for the first two fixations and fixations eight and nine. Whilst more conservative, positive correlations between rightward gaze biases and age were found for fixations three and four with the bootstrapping approach as well (Fig. 3B), with fixations five, six, and seven around *p* = 0.05.

**Table 1:** Pearson correlation coefficients, associated *p*-values, and confidence intervals for correlations between the average gaze position and age for the horizontal and vertical dimensions

	Horizontal				Vertical			
Fixation	*r*	*p*	95% CI			*r*	*p*	95% CI
1	-0.13	0.330	[-0.37; 0.13]			**0.41**	**0.001**	[0.18; 0.60]
2	0.20	0.118	[-0.05; 0.44]			**0.64**	**< 0.001**	[0.46; 0.77]
3	**0.43**	**0.001**	[0.20; 0.62]			**0.79**	**< 0.001**	[0.67; 0.87]
4	**0.42**	**0.001**	[0.19; 0.61]			**0.68**	**< 0.001**	[0.52; 0.80]
5	**0.33**	**0.010**	[0.08; 0.54]			**0.63**	**< 0.001**	[0.44; 0.76]
6	**0.29**	**0.025**	[0.04; 0.51]			**0.48**	**< 0.001**	[0.25; 0.65]
7	**0.31**	**0.019**	[0.06; 0.52]			0.06	0.641	[-0.20; 0.31]
8	0.08	0.559	[-0.18; 0.32]			0.05	0.701	[-0.21; 0.30]
9	-0.02	0.854	[-0.23; 0.28]			-0.12	0.348	[-0.37; 0.13]

*Note* Pearson correlation coefficients and associated *p*-values for tests between age and gaze biases per fixation (rows) for horizontal (left columns) and vertical directions (right columns)

Furthermore, higher age was consistently associated with a more superior gaze position for fixations one to six (see Table 1 for full statistics and Fig. 4A for scatterplots per fixation), peaking at fixation three with a strong effect size (*r* = 0.79, *p* < 0.001). Fixations seven to nine did not show such an association. Positive correlations were found for the same correlations with the bootstrapping approach (Fig. 4B).

**Fig. 5:**
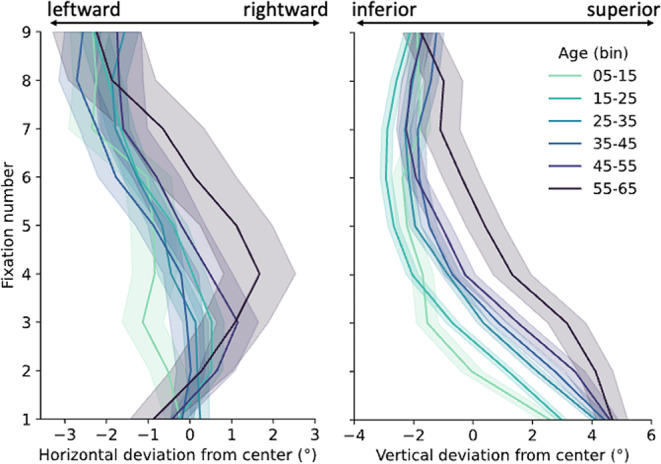
Gaze position deviations relative to the screen center for horizontal (left panel) and vertical (right panel) dimensions over the first nine fixations, binned into ten-year age groups. Note that absolute position deviations (i.e., not age modulations) should not be interpreted, as the stimulus is inherently asymmetric in both dimensions. Shaded areas indicate ± 1 standard error of the mean

Figure 5 visualizes the average gaze positions per fixation across binned age groups (bins of 10 years each). Again, we urge caution before interpreting *absolute* biases here, as the stimulus was not symmetrical across the horizontal or vertical axes, and participants did not necessarily start at the center of the screen. Note that these binned data are visualized for consistency with previous work, but not statistically analyzed.

## Discussion

Horizontal attention biases (‘pseudoneglect) and vertical attention biases (‘altitudinal pseudoneglect’) are thought to reflect cerebral asymmetries in spatial processing. Much work has been devoted to the question of whether spatial attention biases change with age, but results remain inconclusive. Gaze behaviour during free viewing is sensitive to changes in spatial attention and is unaffected by manual or verbal responses and has therefore been suggested as the method of choice for assessing attention biases. Here, fixation data from 4,243 participants, each of whom viewed an image for 10s while their eyes were tracked, revealed consistent effects of aging on spatial attention biases. Specifically, gaze was more rightward and more superiorly biased with increasing age.

We found that the gaze bias became more rightward (or less leftward) with age, which is consistent with the idea of faster right hemisphere ageing (Goldstein and Shelly 1981) and reduced hemispheric asymmetry with age (Cabeza 2002; Cabeza et al. 1997), as well as changes in vertical asymmetries in attention with age (Himmelberg et al. 2023). As both horizontal and vertical biases were affected by aging, this could be seen as tentative support for the idea that cerebral asymmetries drive horizontal and vertical attention biases, at least in that our data are consistent with the *development* of cerebral assymetries with age (Cabeza 2002; Cabeza et al. 1997; Himmelberg et al. 2023). However, it is possible that such cerebral asymmetries develop independently for the horizontal and vertical dimensions, explaining why horizontal and vertical biases have not previously been found to be correlated (Churches et al. 2017; Nicholls et al. 2004; van Vugt et al. 2000; but see Chieffi et al. 2019). Due to the correlational analysis and no direct measure of cerebral asymmetry, however, we cannot exclude the possibility that a factor other than cerebral asymmetry leads to the modulations reported here.

The association between gaze bias and age was found for the first three to seven fixations in the horizontal dimension, and for the first to sixth fixations in the vertical dimension. Interestingly, previous studies have shown that in free viewing, a leftward bias is typically exhibited in these early fixations (Chiffi et al. 2021; Foulsham et al. 2018; Hartmann et al. 2019; Nuthmann and Clark 2023; Nuthmann and Matthias 2014; Ossandon et al. 2014). Such a gaze bias shortly after stimulus onset suggests that spatial attention is not biased per se, but that there is a bias in the spatial *orienting of attention* (as understood based on Petersen and Posner 2012 and Posner 1990). In turn, our findings suggest a modulation of orienting to novel stimuli with age (i.e., the activation-orientation hypothesis; Bultitude and Aimola Davies 2006). Taking this a step further, we speculate that the aforementioned cerebral asymmetries may predominantly affect the orienting of spatial attention in response to novel stimuli, rather than sustained spatial attention, which may be driven less by orienting and visual salience, but more by the goals and interests of the observer. This may in turn explain inconsistencies between tasks (Learmonth et al. 2015a 2015b;Märker et al., 2019; Mitchell et al. 2020; Nicholls et al. 1999), as the importance of attentional orienting may differ between tasks and is crucially affected by the respective time interval of interest.

The temporal dimension of (altitudinal) pseudoneglect has implications for neuropsychological testing of neglect using eye-tracking, which has shown promising diagnostic properties (Cox and Aimola Davies 2020; Müri et al. 2009; Ptak et al. 2009). First, we argue that the assessment of gaze patterns in neglect can be improved by separately analysing the first 1.5s of free viewing rather than averaging horizontal and vertical gaze positions over longer viewing durations. Second, age-matched control groups are of crucial importance given the clear modulations of spatial attention biases in healthy aging presented here. The stimulus used in the present study is freely available via OSF and is currently being used in several other neuropsychological studies. The use of a single free viewing image in neuropsychological testing would add only a few seconds to any eye-tracking test battery. We aim to build a dataset that has sufficient normative data for all age groups.

Although age-related modulations of horizontal and vertical gaze biases were strong in our data, it is important to note that these biases are characterized by large inter-individual variation. This also means that changes in pooled group averages should not be overinterpreted as being deterministic for individuals. Longitudinal rather than cross-sectional data would allow a better understanding of these differences and possible causes for different developmental trajectories.

The data presented here suggest a modulation of attention biases with age that is well captured by a linear relationship. We show here that age effects on horizontal and vertical attention biases are present in the range of 5 to 65 years. These effects are thus seen before accelerated aging after the 60’s, which is often used as a lower bound for extreme group comparisons (Friedrich et al. 2018; Learmonth and Papadatou-Pastou 2021), suggesting more gradual changes in cortical asymmetries with age. However, relatively few participants older than 50 years and too few participants older than 65 years for analyses leave unanswered whether this trend continues linearly, reverses, or even accelerates at older ages, as previously suggested based on different measures (Friedrich et al. 2018; Schaie 1994).

Our setup allowed us to include a uniquely large sample size, but came with a number of limitations. First, the presented image was not symmetrical with respect to the image content. Therefore, we cannot say anything about the *absolute* direction of gaze bias and how it changes with age. Second, we used a low-cost eye-tracker that was installed in a public space, which resulted in lower data quality as compared to more controlled laboratory settings. Nevertheless, the data quality was sufficient (see also Strauch et al. 2023, for a more detailed description). Despite our efforts to control the head position, it could be that some children had their eyes lower than adults. However, as the monitor was positioned downward rather than upward relative to the opening in the metal box, this should be associated with more *upward* gaze positions for children whereas we observed the opposite - more downward gaze positions for younger participants. Third, we did not collect health-related data such as the presence of neurological diseases, and therefore cannot be sure that all participants were neurologically healthy. However, if data from participants with brain damage were driving the effects, this would show up as disproportionately stronger modulations in the oldest participants. In contrast, our data suggest a similar modulation of biases already in younger age groups, where participants are less likely to have suffered brain damage. Furthermore, some self-selection bias is conceivable in that participants had to be sufficiently healthy to come to the museum and complete the data assessment procedure. As a result, the participants providing data here may be *less* rather than more affected by aging overall, suggesting that the effects presented here may be an underestimate of age-related modulations of spatial attention.

Future work will need to show how the changes in spatial attention biases with age described here relate to other measures. As gaze-based assessments are relatively task-free, such associations may be higher than between different task-based measures, which suffer from low inter-task correlations (Learmonth et al. 2015; Märker et al. 2019; Mitchell et al. 2020; Nicholls et al. 1999).

### Electronic supplementary material

Below is the link to the electronic supplementary material.

Supplementary Material 1

## Declarations

### Open practices statement

Compiled data and analysis scripts are available via the Open Science Framework: https://osf.io/3dsr5/. Original data and preprocessing scripts are available via https://osf.io/sk4fr/ (Strauch et al. 2023). This study was not preregistered.
