# Supplementary material for: Gaze data of 4243 participants shows link between leftward and superior attention biases and age
Source: Exp Brain Res. 2024 Mar 31;242(6):1327–37. doi: 10.1007/s00221-024-06823-w (PMC11108882; doi:10.1007/s00221-024-06823-w)
Supplement: Supplementary file 1 — Supplementary Material 1 [file 221_2024_6823_MOESM1_ESM.docx]

**Supplementary Material to: Gaze data of 4243 participants shows link between leftward and superior attention biases and age**

Christoph Strauch^1^**^†^**, Alex J. Hoogerbrugge^1^, Antonia F. Ten Brink^1^**^†^**

^1^Utrecht University, Experimental Psychology, Helmholtz Institute, 3584CS Utrecht, the Netherlands

**^†^These authors contributed equally to this work.**

***Supplementary Figure 1***. Data quality across age bins. Precision in RMS (sample-to-sample root mean square of gaze displacement) and data loss as percentage of lost samples.

***Supplementary Figure 2***. Distributions of correlations over 10,000 bootstraps for 20 participants per year of age (drawn with replacement) for original data (purple solid line) and for data with randomly shuffled labels (turquoise solid line). Vertical dashed lines indicate r = 0. **A:** Distributions for horizontal gaze biases. **B:** Distributions for vertical gaze biases.
